# Supplementary material for: Modeling glioblastoma heterogeneity as a dynamic network of cell states
Source: Mol Syst Biol. 2021 Sep 16;17(9):e10105. doi: 10.15252/msb.202010105 (PMC8444284; doi:10.15252/msb.202010105)
Supplement: Supplementary file 6 — Source Data for Figure 5 [file MSB-17-e10105-s004.zip › Figure5A_sourcedata/GSEA_3017/nosigdb_stateA.GseaPreranked.1622795026485/SUVA_G2M_PMID_31327527.html]

Details for gene set SUVA\_G2M\_PMID\_31327527[GSEA]

|  || Dataset | state53017 |
| Phenotype | NoPhenotypeAvailable |
| Upregulated in class | na\_pos |
| GeneSet | SUVA\_G2M\_PMID\_31327527 |
| Enrichment Score (ES) | 0.534055 |
| Normalized Enrichment Score (NES) | 2.7908838 |
| Nominal p-value | 0.0 |
| FDR q-value | 0.0 |
| FWER p-Value | 0.0 |
Table: GSEA Results Summary

  

Fig 1: Enrichment plot: SUVA\_G2M\_PMID\_31327527      
 Profile of the Running ES Score & Positions of GeneSet Members on the Rank Ordered List

  

| PROBE | GENE SYMBOL | GENE\_TITLE | RANK IN GENE LIST | RANK METRIC SCORE | RUNNING ES | CORE ENRICHMENT || 1 | KIF11 |  |  | 6 | 0.870 | 0.0652 | Yes |
| 2 | KPNA2 |  |  | 20 | 0.667 | 0.1065 | Yes |
| 3 | CCNB1 |  |  | 37 | 0.599 | 0.1390 | Yes |
| 4 | CCNA2 |  |  | 65 | 0.495 | 0.1515 | Yes |
| 5 | AURKA |  |  | 78 | 0.474 | 0.1779 | Yes |
| 6 | CDC20 |  |  | 80 | 0.472 | 0.2156 | Yes |
| 7 | PLK1 |  |  | 89 | 0.464 | 0.2454 | Yes |
| 8 | TUBB4B |  |  | 93 | 0.462 | 0.2802 | Yes |
| 9 | CENPF |  |  | 94 | 0.461 | 0.3181 | Yes |
| 10 | CDCA8 |  |  | 115 | 0.437 | 0.3331 | Yes |
| 11 | BUB1B |  |  | 129 | 0.421 | 0.3541 | Yes |
| 12 | CCNB2 |  |  | 147 | 0.411 | 0.3702 | Yes |
| 13 | KIFC1 |  |  | 160 | 0.402 | 0.3907 | Yes |
| 14 | ARHGAP11A |  |  | 162 | 0.399 | 0.4225 | Yes |
| 15 | KIF2C |  |  | 165 | 0.397 | 0.4530 | Yes |
| 16 | BUB1 |  |  | 188 | 0.384 | 0.4616 | Yes |
| 17 | CDCA3 |  |  | 203 | 0.372 | 0.4776 | Yes |
| 18 | SMC4 |  |  | 217 | 0.367 | 0.4941 | Yes |
| 19 | H2AFZ |  |  | 245 | 0.351 | 0.4948 | Yes |
| 20 | TTK |  |  | 262 | 0.340 | 0.5061 | Yes |
| 21 | KNSTRN |  |  | 263 | 0.340 | 0.5341 | Yes |
| 22 | TUBA1C |  |  | 322 | 0.318 | 0.4996 | No |
| 23 | ECT2 |  |  | 365 | 0.305 | 0.4808 | No |
| 24 | CKAP2 |  |  | 379 | 0.301 | 0.4920 | No |
| 25 | RACGAP1 |  |  | 399 | 0.293 | 0.4962 | No |
| 26 | NCAPD2 |  |  | 440 | 0.283 | 0.4778 | No |
| 27 | KIF20A |  |  | 447 | 0.281 | 0.4946 | No |
| 28 | ANP32E |  |  | 453 | 0.280 | 0.5124 | No |
| 29 | KIF4A |  |  | 504 | 0.269 | 0.4824 | No |
| 30 | TMPO |  |  | 584 | 0.253 | 0.4207 | No |
Table: GSEA details [plain text format]

  

Fig 2: SUVA\_G2M\_PMID\_31327527: Random ES distribution      
 Gene set null distribution of ES for **SUVA\_G2M\_PMID\_31327527**
